# Spectroscopic analysis of the bacterially expressed head domain of *rotavirus* VP6

**DOI:** 10.1042/BSR20232178

**Published:** 2024-04-29

**Authors:** Milaan Simone Strachan, Tshepo Mashapa, Samantha Gildenhuys

**Affiliations:** Department of Life and Consumer Sciences, College of Agriculture and Environmental Sciences, University of South Africa, Private Bag X6, Florida, Roodepoort 1710, South Africa

**Keywords:** far-UV circular dichroism, IMAC chromatography, intrinsic tryptophan fluorescence spectroscopy, rotavirus, stability, VP6 head domain

## Abstract

The rotavirus capsid protein VP6 forms the middle of three protein layers and is responsible for many critical steps in the viral life cycle. VP6 as a structural protein can be used in various applications including as a subunit vaccine component. The head domain of VP6 (VP6_H_) contains key sequences that allow the protein to trimerize and that represent epitopes that are recognized by human antibodies in the viral particle. The domain is rich in β-sheet secondary structures. Here, VP6_H_ was solubilised from bacterial inclusion bodies and purified using a single affinity chromatography step. Spectral (far-UV circular dichroism and intrinsic tryptophan fluorescence) analysis revealed that the purified domain had native-like secondary and tertiary structures. The domain could maintain structure up to 44°C during thermal denaturation following which structural changes result in an intermediate forming and finally irreversible aggregation and denaturation. The chemical denaturation with urea and guanidinium hydrochloride produces intermediates that represent a loss in the cooperativity. The VP6_H_ domain is stable and can fold to produce its native structure in the absence of the VP6 base domain but cannot be defined as an independent folding unit.

## Introduction

Rotaviruses (RVs) are the etiological agent of gastroenteritis, a diarrhoeal disease with a high morbidity and mortality rate, particularly in infants and young children [1–4]. Due to their prevalence and impact on public health, understanding and managing RV infections is crucial for effective prevention and treatment strategies. The current strategy for RV management mostly relies on the administration of live attenuated vaccines but variation exists in the efficacy and thus identifying viable alternatives will be valuable.

The RV replication/infection cycle involves conformational changes of protein building blocks of the virus capsid, facilitating virion transition from an infectious triple-layered particle (TLP) form to a transcriptionally active double-layered particle (DLP) form. A mature infectious RV virion (TLP) describes a nonenveloped, icosahedral assembly of three distinct protein layers concentrically arranged to encapsulate the RV nucleoprotein core (reviewed in references [1–4]). The outermost layer comprises structural proteins VP4 and VP7 which facilitate binding, entry, and uncoating [5,6]. The intermediate layer consists exclusively of VP6 trimers that maintain the structural integrity of the double- and triple-layered viral particles and activate viral transcription upon loss of the outermost layer [1,7,8]. It has been shown that VP7 and VP6 associate in the absence of other viral proteins [9] and that VP6 interacts with one of the non-structural glycoproteins NSP4 [10]. The structural protein VP2 forms the shell of the viral core and encloses the double-stranded RNA genome as well as the RNA-dependent RNA polymerase (VP1) and mRNA capping enzyme (VP3) [11] complexes.

VP6 has long been an attractive candidate for the development of novel anti-RV therapeutics given its well-established immunogenic nature and the high structural conservation across known strains. The conservation suggests a highly evolved structure fit for purpose [12], making VP6 a promising target for the development of broad-spectrum antiviral drugs and subunit vaccines that protect against multiple RV strains [1,7,9]. VP6-based RV therapeutics may induce both cross-reactive and homologous immune responses [7,12,13]. VP6 can also form single-layered viral particles without trimerization [14]. Furthermore, several studies have reported successful use of this protein in various biotech applications, such as an adjuvant [15,16], vaccine [17–20], drug delivery system [21–24], as well as scaffolds for the construction of nano-biomaterials [25–28]. For many of the potential applications, a simplified version of VP6 or the head domain alone could potentially act in the roles.

The structure of a VP6 monomer is composed of a β-sheet head (VP6_H_) domain and an α-helical base (VP6_B_) domain [1]. The VP6_H_ domain is known to form direct contact with the outermost layer of the viral capsid [1,11,29] and contains the key residues needed for trimerization [1,30]. Furthermore, the domain also contains epitopes that are recognised by human antibodies and is, therefore, responsible for immunogenic properties of VP6 [7,11,13,31,32]. Through this domain, the loss of the outer layer is communicated to the viral polymerase (VP1) a further layer below the VP6 middle layer of the viral capsid [1,7,8]. Therefore, a network of interactions exists within the protein to convey structural signals and facilitate conformational changes.

The most simplistic version of protein folding involves only the native and unfolded states and a cooperative transition from one to the other. For many proteins the presence of intermediates further complicates the process introducing more steps and reducing cooperativity. Cooperative folding is associated with key interactions taking place between distinct amino acid residues within a protein [33]. If you reduce the chain length of a protein this can interfere with the interaction network and disrupt key packing requirements which can lead to less cooperative folding [34]. Protein domains have been viewed as independent folding units [35], although one can’t ignore that long-range tertiary contacts influence protein folding [36] and hence could influence the folding of multidomain proteins.

VP6_H_ domain has previously been expressed, purified and could form native structures [37] indicating the domain could form independently from the VP6_B_ domain. We sought to prepare the VP6_H_ domain (from a consensus sequence of the VP6 protein [12]) to assess its secondary and tertiary structure as well as stability in the absence of the VP6_B_ domain for use in various applications. Our findings indicate that although the VP6_H_ domain can independently obtain its native secondary and tertiary structure, this is not done in a cooperative manner. An interaction network spanning VP6 must be responsible for the dynamic structural nature of the protein. The interactions network within the full-length protein likely guides specific conformational changes to occur as required in the viral life cycle.

## Materials and methods

### Materials

Competent *Escherichia coli* BL21 (DE3) and NiCo21 (DE3) cells were purchased from New England Biolabs (Massachusetts, United States of America). The pET15a plasmid containing the codon optimised consensus sequence of cDNA that encodes VP6 residues 147 - 332 (MGFVFHKPNIFPYSASFTLNRSQPMHDNLMGTMWLNAGSEIQVAGFDYSCAINAPANIQQFEHIVQLRRALTTATITLLPDAERFSFPRVINSADGATTWFFNPVILRPNNVEVEFLLNGQIINTYQARFGTIIARNFDTIRLSFQLMRPPNMTPAVNALFPQAQPFQHHATVGLTLRIESAVCES [12]) was purchased from GenScript (Shanghai, China) The His-Trap HP columns were purchased from Cytiva (Massachusetts, United States of America). Isopropyl ß-D-1-thiogalactopyranoside (IPTG) and ampicillin were purchased from Glentham Life Sciences (Corsham, United Kingdom). High-purity urea was purchased from Melford (Ipswich, United Kingdom). All other reagents used in this study were of analytical grade.

### Methods

#### Expression

Competent *E. coli* BL21 (DE3) and NiCo21 (DE3) cells were transformed with the pET15a plasmid containing the bacterial codon-optimised cDNA consensus sequence of VP6_H_. A starter culture was prepared for each strain by inoculating a colony from each strain into sterile LB broth supplemented with 100 μg/ml ampicillin and grown overnight at 37°C with shaking at 200 rpm. The overnight cultures were diluted 50 times into sterile LB broth supplemented with ampicillin and incubated as for the starter culture until growth produced an OD_600_ of 0.6 which was shown to represent early-log phase. Protein expression was induced for BL21 (DE3) cultures with either 0.1 mM or 1.0 mM IPTG and for NiCo21 (DE3) cultures with 0.04 mM and 0.4 mM IPTG. The cultures were grown for 2–16 h at 37°C and at 20°C overnight and collected by centrifugation. Cells were resuspended in ultra-pure water and lysed by sonication. The lysates were centrifuged (13400 rpm for 25 min) and the supernatant and pellets were analysed by SDS-PAGE.

#### Solubilisation

A single freeze-thawing cycle method described by Qi and colleagues [38] was employed in the solubilisation study. The lysate pellet was washed once in 100 mM Tris-HCl pH 7.00 with 1% Triton X-100 then twice in 100 mM Tris-HCl pH 7.00 to remove the detergent. Samples were centrifuged between washes at 13400 rpm for 15 min. The washed pellets were resuspended in 100 mM Tris-HCl of various pH values (7.00–10.00) [38] and urea concentrations (0, 2, and 5 M). The samples were incubated at room temperature for 90 min before being frozen at −20°C overnight. The frozen samples were thawed at room temperature [38], centrifuged at 15000 rpm for 15 min, and the supernatants and pellets were analysed by SDS-PAGE.

#### Protein purification and quantification

VP6_H_ purification was performed employing IMAC using a commercially available pre-packed 5 ml nickel ion (Ni^2+^) charged affinity column (Cytiva, Massachusetts, U.S.A.). The protein content of the mobile phase flow-through was monitored by recording A280 in all steps of the experiment. The solubilised IBs protein was applied onto a column which was pre-equilibrated with 100 mM Tris-HCl pH 9.00, 2 M urea, 150 mM NaCl, 40 mM imidazole, and 0.02% (w/v) sodium azide using a Bio-Rad (California, United States of America) NGC™ Chromatography system. The column was then washed with 100 mM Tris-HCl pH 9.00, 2 M urea, 300 mM NaCl, 40 mM imidazole, and 0.02% (w/v) sodium azide. Bound protein was eluted using a 40–600 mM imidazole concentration gradient and collected in 2 ml fractions which were analysed by SDS-PAGE. Fractions containing pure VP6_H_ were pooled and dialysed into 20 mM sodium phosphate buffer pH 7.40 with 0.02% (w/v) sodium azide.

The concentration of the purified protein was determined by absorbance spectroscopy and Bradford assay. Absorbance spectroscopy was done in a 10 mm quartz cuvette using the Applied Photophysics Chirascan Plus (Leatherhead, United Kingdom) with a bandwidth of 0.5 nm and step size of 1 nm. The spectra were recorded in triplicate between 220 and 340 nm. After buffer contributions had been subtracted, the concentration of the sample was calculated using the Beer-Lambert law (*A* = ε*l*[*C*] where the extinction coefficient, ε, was calculated using the equation described by Pace et al. [39]. In the present study, the value of ε is 15720 M^−1^. cm^−1^. A Bradford assay, using bovine serum albumin, was also done to verify the concentration of the sample. The colour change in the standards and sample was monitored by measuring the absorbance at 595 nm using the Implen P330 Nanophotometer (München, Germany).

#### Spectral characterisation

A band from the purification SDS-PAGE gel was excised and sent to the Council of Scientific and Industrial Research (CSIR) (Pretoria, South Africa) where the protein was extracted from the gel and cleaved using trypsin [40]. The peptides thus obtained were analysed by mass spectroscopy (LSMS) using a Dionex Ultimate 3000 RSLC system coupled to an AB Sciex 5600 TripleTOF mass spectrometer.

Far ultraviolet circular dichroism (far-UV-CD) and intrinsic tryptophan fluorescence were conducted using the Applied Photophysics Chirascan Plus (Leatherhead, United Kingdom). A concentration of 10 μM VP6_H_ was used for all far-UV-CD and intrinsic tryptophan fluorescence spectra. The far-UV-CD spectra and voltage (HV) were recorded between 200 and 250 nm in a 1 mm quartz cuvette with a bandwidth and step size of 2 nm and 1 nm, respectively. The secondary structure composition was quantified using the K2D3 tool [41] available at http://cbdm-01.zdv.uni-mainz.de/∼andrade/k2d3/ after converting the raw data to mean residue ellipticity using the following formula: [θ] = 100 (signal) /Cnl. Where the signal is the far-UV-CD reading in millidegrees (mdeg); c is the millimolar (mM) concentration of the protein; n is the number of amino acids; and l is the pathlength in cm.

Intrinsic tryptophan fluorescence spectroscopy was conducted in a 10 mm quartz cuvette fluorescence emission spectra were recorded between 240 and 500 nm following excitation at 295 nm. The bandwidth and step size used were 2.5 and 1 nm, respectively.

#### Molecular dynamics

The protein structures were generated using AlphaFold [42] which was accessed through an open-source Google colab [43]. The consensus sequence was entered, the program was run according to the developers’ instructions, and a generated model was selected. The monomeric and trimeric structures thus obtained were then prepared for molecular dynamics using the default prepare protein settings (build loops and protonate at pH 7.4) in Discovery Studio client v20.1.0.19, followed by assigning a charmm36 forcefield as well as solvating the protein. The solvation model used was the explicit periodic boundary and sodium chloride at a concentration of 0.145 M was introduced. The standard dynamics cascade was conducted using default settings other than in the equilibration step a simulation time of 20 ps was selected, as well as in the production step observation was altered to 200 ps. Following the simulation the trajectories of the conformations generated were analysed to produce Root Mean Squared Fluctuation (RMSF) values for each amino acid which represent an average for all the heavy atoms making up each amino acid.

#### Chemical stability

Ten molar (10 M) urea and 6 M guanidine hydrochloride (GdCl) stock solutions were prepared according to the methods of Pace [44] using 20 mM sodium phosphate buffer pH 7.40. The protein was left to equilibrate in 0.5–8 M urea and 0.25–5 M guanidine hydrochloride for 3 h at room temperature before recording spectra for each sample. To test the reversibility of chemical unfolding, the domain was treated with buffers containing 8 M urea and 5 M guanidine hydrochloride, respectively, and left to equilibrate for 3 h at room temperature. Thereafter, the samples were diluted using 20 mM sodium phosphate buffer pH 7.40 and kept at room temperature for 3 h to equilibrate before far-UV-CD and fluorescence spectra were recorded (as previously described). These spectral data were converted to fraction unfolded using the following formula: *F*_u_ = (*y* −  *y*_N_)/(*y*_D_ −  *y*_N_), where *y* is the signal obtained from a spectral probe, *y*_N_ and *y*_D_ are the *y* values obtained following the extrapolation of the least squares regression lines fitted to the pre- and post-transition regions respectively. SciDAVis (version 2.7.1) was used to plot all spectral data.

#### Thermal stability

Far-UV-CD and intrinsic tryptophan fluorescence spectroscopy were used (as described previously) to probe changes in the secondary and tertiary structure of VP6_H_ in response to heat-induced denaturation (20–90°C). The temperature was regulated by the PCS.3 Single Cell Peltier Temperature Controller with its accompanying chiller unit (Leatherhead, United Kingdom)

## Results

### Expression in BL21 (DE3) and NiCo21 (DE3) *E. coli* cell lines

The over expression of VP6_H_ in BL21 (DE3) and NiCo21 (DE3) cells was in the insoluble fraction (Supplementary Figure S1). The calculated relative weight of the expressed protein was 20.7 kDa, which is consistent with the theoretical weight of 20.79kDa calculated using ProtParam tool on ExPASy [45] (Supplementary Figure S1). Both cell lines produced comparable amounts at the low IPTG concentrations and there was no substantial increase after 7 h (Supplementary Figure S1). Reducing the expression temperature to 20°C did not produce protein in the soluble fraction (data not shown).

### Solubilisation

NiCo21 (DE3) cells induced with 0.04 mM IPTG and grown for 7 h post-induction at 37°C were lysed, and the pellets washed (Supplementary Figure S2A) before being resuspended in Tris-HCl buffers that varied in pH [37] (7.00–10.00) and urea concentrations (0, 2, and 5 M). This was followed by a single freeze-thaw step [46]. The SDS-PAGE gel of the solubilisation study (Supplementary Figure S2B–E) shows that VP6_H_ remained insoluble in the absence of urea regardless of pH and insoluble at pH values below 9 regardless of urea concentration indicating that pH aids solubility but does not exclusively influence it. Comparable solubilisation is achieved in pH 9 and pH 10 solutions with at least 2 M urea, however, there is less insoluble protein remaining in pH 9. Therefore, the solubilisation conditions selected involved resuspending the pellet containing insoluble VP6_H_ in 100 mM Tris-HCl pH 9.00 with 2 M urea, freezing at −20°C, and thawing at room temperature.

### Purification and protein quantification

Soluble VP6_H_ was purified by His-tag affinity chromatography using a nickel resin. The protein was eluted using an imidazole gradient and eluted in nine 2 ml fractions between 150 and 270 mM imidazole (Supplementary Figure S3A). SDS-PAGE analysis indicated protein fractions to be of high purity (Supplementary Figure S3B). Purified VP6_H_ concentration was found to be 0.95 and 0.93 mg/ml based on the absorbance spectra and Bradford assay, respectively. This corresponds to approximately 13.32 mg of pure protein from 1 L of culture.

### Protein characterisation

**Figure 1:**
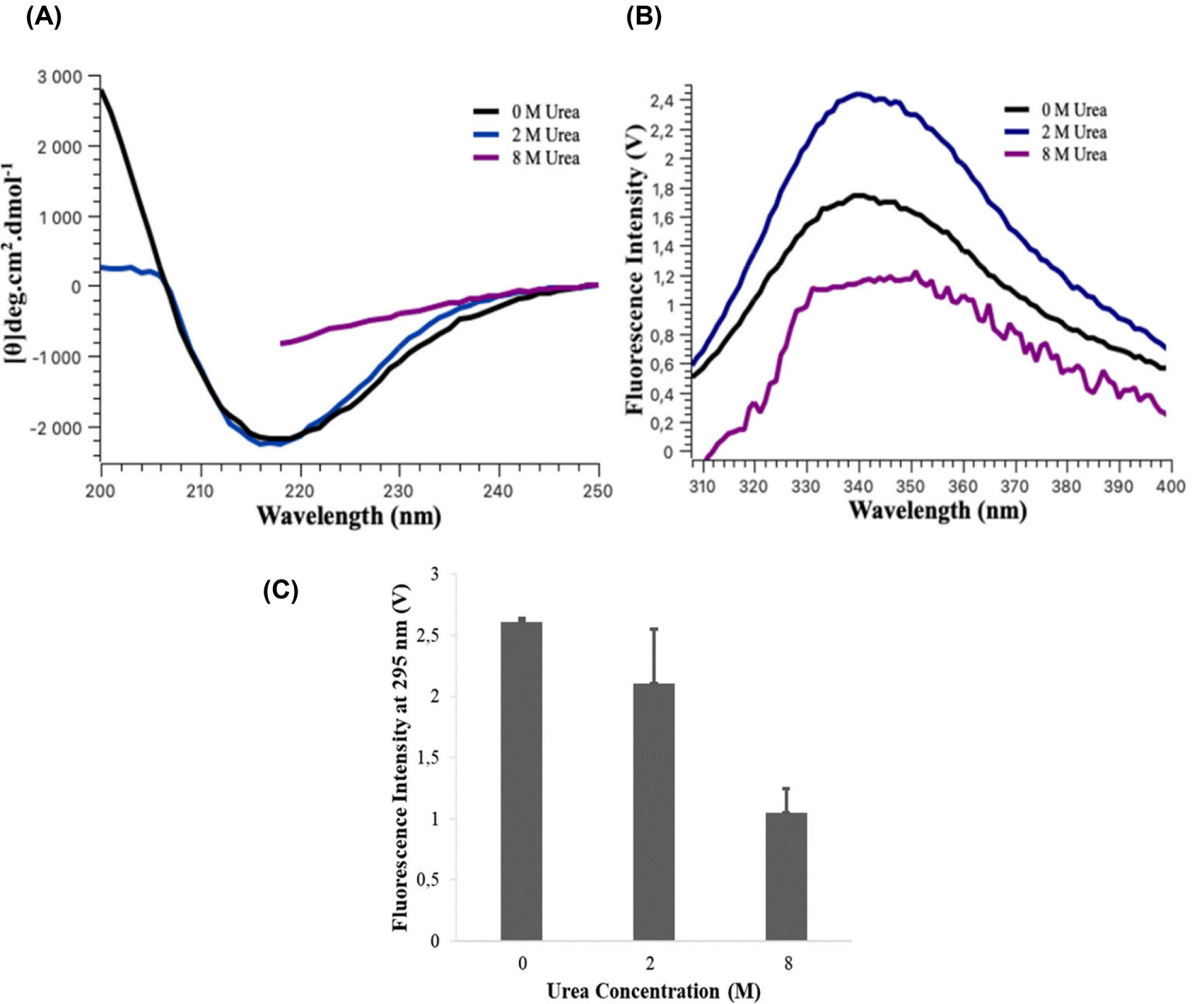
Characterisation of VP6H in the presence and absence of urea VP6_H_ in 0, 2, and 8 M urea (**A**) Far-UV-CD spectra (**B**) Intrinsic fluorescence spectra and (**C**) Fluorescence emission at 295 nm or scatter of excitation wavelength.

The mass spectrometry results confirmed that the purified protein was VP6_H_. The mass spectrometry data also showed that one asparagine residue (Asp158 or 305 in the full-length VP6) was deamidated of a total of 26 asparagine and glutamine residues which could be a result of the pH of the buffer used during solubilisation and purification. Asparagine residues are more vulnerable to deamidation when located next to a residue with a small side-chain group on its C-terminal end, as there is no steric hindrance that shields it from deamidation [47]. The deamidated asparagine residue has an alanine residue (Ala 159/306) on its C-terminal end. The deamination did not affect the secondary structure of VP6_H_, which contains 48% β-sheet structures as calculated from the AlphaFold structure model. Far-UV-CD spectra of the domain in the absence of urea revealed a negative band at 218 nm and a positive band at 200 nm and in 2 M urea, the magnitude of the negative band is maintained; however, there is a decrease in the magnitude of the positive band at 200 nm (Figure 1A). This decrease is attributed to a decrease in the signal-to-noise ratio as an increase in the sample turbidity (HV) occurs in this region due to the presence of the urea. Quantification of the spectra on K2D3 [41] revealed that the protein contains 46% and 44% β-sheet structures in 0 and 2 M urea, respectively which further confirm the domain has native-like secondary structures even with the addition of 2 M urea. In 8 M, urea the negative band at 218 nm is reduced indicating loss of secondary structure.

The intrinsic tryptophan residues (Trp34 and Trp100 or 181 and 247 in the full-length VP6) were used to probe the tertiary structure of the domain in the presence and absence of urea (Figure 1B). The maximum emission wavelength (λ_max_) was at 338 nm in 0 and 2 M urea, indicating that the tryptophan residues were mostly buried in both conditions [48]. There was, however, an increase in the fluorescence intensity of the sample in 2 M urea. In 8 M urea the emission wavelength (λ_max_) was at 351nm, indicating increased exposure of tryptophan residues to solvent [48]. Overall, intrinsic tryptophan fluorescence suggests VP6_H_ to assume a native-like tertiary structure under both mild and non-denaturing conditions, which is expectedly lost under denaturing conditions. Figure 1C shows the intensity of the scattered excitation light, the 0 M urea sample produces the greatest scatter which decreases as the urea concentration increases. This means that there is a reduction in the number of larger structures that form in the presence of urea.

### Molecular dynamics

**Figure 2:**
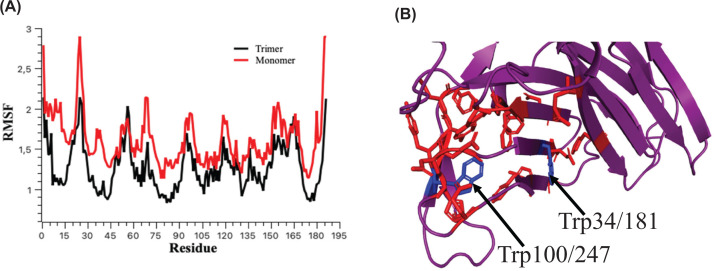
Standard Molecular dynamics simulation of VP6_H_ (**A**) RMSF values are average values for all heavy atoms of VP6H residues in the trimer (black) and monomer (red). (**B**) Tryptophan residues (blue sticks) and surrounding quenching amino acids (red sticks).

AlphaFold generated five models for the β-domain monomer, when aligning these models, the only variation occurred in the region Ile91- Trp100 (Ile238- Trp 247 in the full-length protein) for one of the five models (Supplementary Figure S4), where the secondary structure was maintained but the tertiary arrangement differed. One of the four identical models was then used for the standard dynamics simulation. The secondary structure of the VP6_H_ monomer remains reasonably constant in the 100 different conformations generated. The only changes to the secondary structure occur in the region of Glu83 - Ser86 (230-233 in full-length VP6), where a transition is made from a loop to a helical structure. There are no major changes in RMSF for both monomer and trimer and they are on average below 1.61 and 1.27, respectively (Figure 2A) although the residues within the trimer understandability show lower flexibility than in a monomer structure. The residues near Trp34 and Trp100 (181 and 247 in the full-length VP6) as shown in Figure 2B, include several Phe (17/164, 101/248, and 102/249), His (26/173), Asn (28/175, 92/239, 95/242), Asp (27/174), Gln (22/169), and Tyr (48/195) residues which are known to quench tryptophan fluorescence by various mechanisms [49]. The tryptophan fluorescence intensity increased in the presence of 2 M urea (Figure 1B) which could be due to changes in the protein quaternary structure and hence flexibility in the residues surrounding the tryptophan residues as has been shown in other proteins [50].

### Protein thermal stability

**Figure 3:**
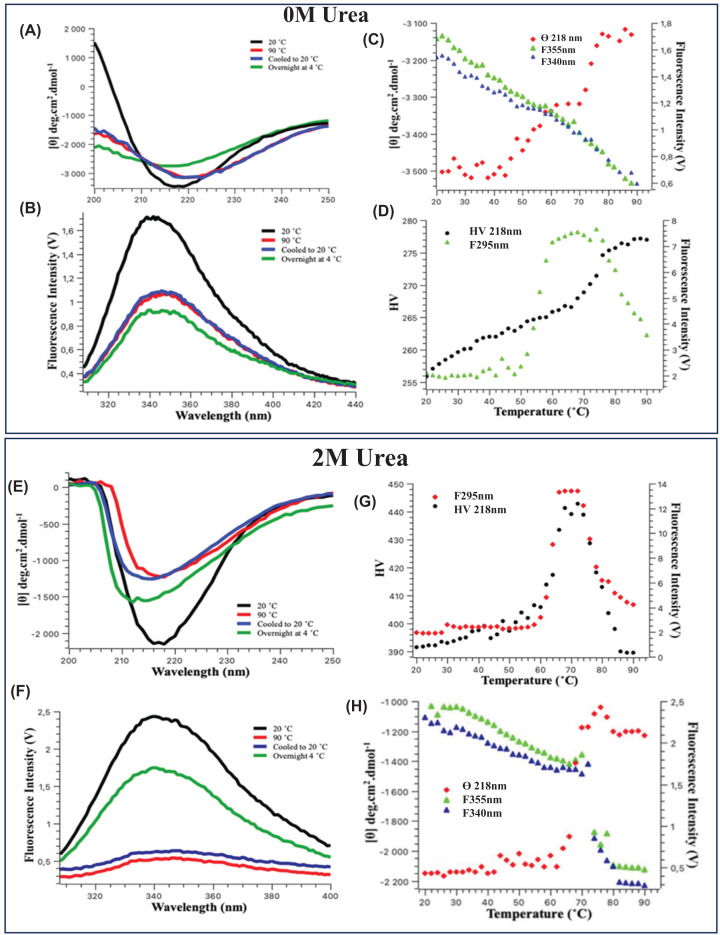
Thermal unfolding of VP6_H_ (**A,E**) Far-UV CD spectra in 0 and 2 M urea before heating and after cooling from 90°C to 20°C or overnight at 4°C. (**B,F**) Intrinsic fluorescence spectra in 0 and 2 M urea before heating and after cooling from 90°C to 20°C or overnight at 4°C. (**C,H**) Plot of the ellipticity at 218 nm and fluorescence intensity at 340 and 355 nm in 0 and 2 M urea. (**D,G**) Plot of the sample turbidity (HV) at 218 nm and fluorescence intensity at 295 nm in 0 and 2 M urea.

The thermal stability of VP6_H_ was characterised by spectroscopy in 0 and 2 M urea. The far-UV-CD spectra of the 0 M urea sample (Figure 3A) shows slight increases in ellipticity between 210 and 250 nm and a significant decrease in ellipticity between 200 and 210 nm when heated to 90°C indicating a loss in the secondary structure of the protein. The sample left to cool to 20°C and overnight at 4°C does not regain the native secondary structure, therefore VP6_H_ cannot be recovered after thermal denaturation. The fluorescence spectra of the 0 M urea sample (Figure 3B) also indicated that tertiary structure is not regained after cooling from 90°C to 20°C or overnight at 4°C.

To follow the changes in the secondary structure with temperature the ellipticity at 218 nm values were plotted and the tertiary structural changes represented by fluorescence intensity at 340 and 355 nm (Figure 3C). The trend displayed by the ellipticity values shows structural changes from 44°C which until 60°C resulting in reduced ellipticity at 218 nm. Then there are limited changes until 68°C where we see a rapid reduction in the 218 nm values until 79°C. This pattern may be indicative of an intermediate structure that forms as the protein is heated. There are no corresponding observed patterns in the intrinsic tryptophan fluorescence values at 340 nm, representing the folded emission maximum, and the 355 nm values representing the unfolding emission maximum (Figure 3C). The fluorescence intensity decreases at both wavelengths as the protein is heated. This trend most likely results from the conformational rearrangement of the tryptophan residues during heating. It could also be due to thermal deactivation of fluorophores. One would also have to consider the scattering of the excitation light by aggregates formed with heat denaturation. The 295 nm wavelength values at corresponding temperatures were plotted (Figure 3D), a distinct increase is observed from 50°C, with a peak in the 60°C to 68°C region corresponding to the intermediate structure detected by far-UV CD. The HV voltage reading taken during circular dichroism measurements indicates the voltage the detector requires to obtain a reading based on the light reaching the detector [51]. If large protein aggregates interfere with the light from the source to the detector, this reading will increase. This phenomenon is visible for VP6_H_ (Figure 3D) as the HV increases with temperature, resulting in a rapid increase from 70°C. The intermediate detected is a structure formed as the protein is aggregating with heating.

In order to potentially prevent the aggregation of VP6_H_, the thermal stability was also characterised in the presence of 2 M urea because urea is known to preferentially interact with hydrophobic regions in proteins [52–54]. Also 2 M urea solubilized the protein, and the structure was shown to be native-like in this concentration of urea.

The protein structure before and after cooling from 90°C to 20°C or overnight at 4°C was monitored using far-UV-CD (Figure 3E) and intrinsic tryptophan fluorescence (Figure 3F). VP6_H_ could not regain secondary and tertiary structure after cooling from 90°C to 20°C or overnight at 4°C and hence the native protein could not be recovered. The fluorescence spectra recorded at 90°C lacks a clear emission maximum peak as the intensity was very low (Figure 3F). Peak trends observed in both 295nm fluorescence excitation scatter as well and CD HV readings were seen starting at 60°C indicating that aggregation was still taking place (Figure 3G). The wavelength trends looking at both secondary and tertiary structures (Figure 3H) show structural changes at approximately 60°C. The presence of urea has removed the formation of an intermediate structure and delayed overall aggregation to higher temperatures followed by unfolding.

### Protein chemical stability

**Figure 4:**
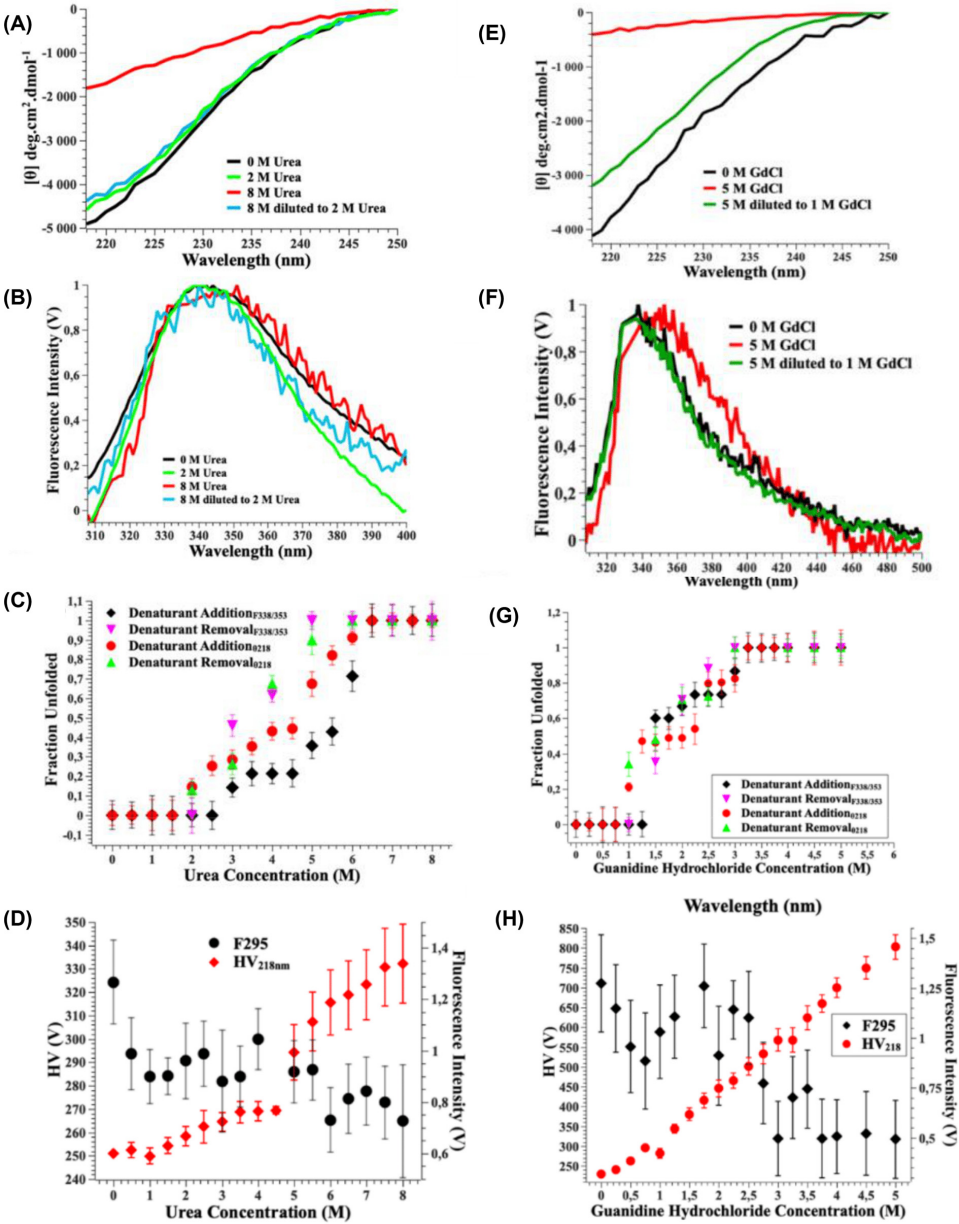
Chemical Unfolding of VP6_H_ Changes to the secondary and tertiary structure VP6_H_ following the addition and removal of urea (**A,B**) and guanidine hydrochloride (**E,F**) were monitored by far-UV-CD and intrinsic fluorescence spectroscopy. Plot of the ellipticity at 218 nm and fluorescence intensity at 340 nm/353 nm for various urea (**C**) and guanidine hydrochloride (**G**) concentrations for the addition or dilution(removal) of the denaturants. Plot of the sample turbidity (HV) at 218 nm and fluorescence intensity at 295 nm for various urea (**D**) and guanidine hydrochloride (**H**) concentrations.

VP6_H_ secondary (Figure 4A,E) and tertiary structure (Figure 4B,F) could be recovered following denaturation with urea and guanidine hydrochloride (GdCl). Therefore, the equilibrium unfolding changes were monitored by far-UV-CD and intrinsic tryptophan fluorescence spectroscopy in the presence of 0–8 M urea (Figure 4C,D) and 0–5 M guanidine hydrochloride (Figure 4G,H). The far-UV-CD spectra were recorded between 218 and 250 nm as this region had the best signal-to-noise ratio with the denaturants present. Similarly, the wavelengths corresponding to the native conformation (338 nm) and denatured conformation (353 nm) were selected from the fluorescence data to visualise changes in tertiary structure. The far-UV-CD and intrinsic tryptophan fluorescence unfolding data obtained from urea-induced unfolding shows a similar trend as measured by the different probes however, the secondary structure appears to start to unfold from a slightly lower urea concentration (2 M) than the tertiary structure (3 M) (Figure 4C). The tertiary structural probe is not global as the two tryptophan residues Trp34/181 and Trp100/247 are located reasonably close to one another within the protein (Figure 2B). Flexibility does not necessarily mean instability, so even if a region is flexible, it can still be stable [55,56]. There may be an intermediate structure that forms when the domain is chemically unfolded as detected with both probes. Fluorescence data indicate that there are no changes in the local environment of the tryptophan residues between 3.5 and 4.5 M urea while far-UV-CD detects secondary structural changes from 3.5 to 4 M, but minimal changes are seen between 4 and 4.5 M urea.

To determine if the unfolding pattern was also reversible, the protein was unfolded in 8 M urea and then the urea concentration was reduced by 1 M in a stepwise manner and intrinsic tryptophan fluorescence and far-UV-CD spectra were recorded. These data show different trends to the unfolding data (Figure 4C). For the fluorescence data VP6_H_ is fully unfolded in 5 M urea, and 50–60% unfolded in 3 M and 4 M urea, respectively. This is not in agreement with the unfolding data which indicates that the domain is only 40% unfolded in 5 M urea and 20% unfolded between 3 and 4 M urea. The far-UV-CD data aligns between 8–6 M and 3–2 M urea. The amount of protein that is unfolded between 5 and 4 M urea differs between the unfolding and refolding transitions indicating the process is not fully reversible even though the protein can be recovered following chemical denaturation with urea.

The fluorescence excitation wavelength scatter data (Figure 4D) shows no increase as the urea concentration increases. Urea affected the signal-to-noise ratio of the samples and hence there should be a linear increase in HV with increasing urea concentration but the increase in the HV at 218 nm increases rather steeply from 5 M urea (Figure 4D). Therefore, aggregation or the presence of large oligomeric species cannot be ruled out as the cause of the irreversible unfolding of the VP6_H_ domain in urea. An off-pathway aggregation reaction could also explain differences observed between unfolding and refolding transitions.

When GdCl is used as a denaturant in both unfolding and refolding studies the trends as monitored by far-UV-CD and intrinsic fluorescence spectra (Figure 4G) aligned more closely indicating reversible unfolding. The unfolding of VP6_H_ in GdCl revealed a different unfolding pathway to that of urea, there are two intermediate structures detected by far-UV CD between 1.25–2 M and 2.5–3 M GdCl and fluorescence spectroscopy between 2.25–2.5 M and then 2.5–2.75 M GdCl. These intermediates do not seem to be due to the formation of larger oligomers or aggregation as there is a steady increase in the HV at 218 nm with increasing denaturant and no increase in the scattered excitation wavelength values at 295 nm in the fluorescence data (Figure 4H). The secondary structure seems to undergo changes before the tertiary structure which is like the urea trend. The transition for GdCl is a complicated pathway involving intermediates.

## Discussion

VP6_H_ has been purified previously and shown to fold to produce a trimeric protein that would interact with antibodies [37]. It is, therefore, not surprising that gentle solubilization conditions were sufficient to solubilize the protein for further purification. The insolubility at pH 7 and pH 8 could also be due to these conditions being too close to the pI of the domain which was computed using ProtParam [45] to be 7.35. Urea was a key component used in solubilization at a concentration that does not denature the protein (Figure 1) but rather interacts with hydrophobic regions decreasing the oligomerization or aggregation tendency. During thermal denaturation the presence of 2 M urea also delayed irreversible aggregation with changes only starting to take place at 60°C (Figure 3). By nature, rotavirus VP6 will contain many surfaces that will interact to form the trimer as well as the viral capsid layer. In fact, VP6 subunit self-assembly into many alternative large structures (such as tubes) of various sizes in response to subtle changes in pH and the presence of divalent ions have been previously detected [57]. VP6 alternative structures may be functional since they have been shown to have applications in vaccine development and drug delivery [20].

Protein stability is key in the development and evaluation of protein-based therapeutics such as vaccines. VP6 is an attractive candidate for the development of novel rotavirus vaccines due to the beta-sheet domain-bearing epitopes that are recognized by human antibodies [7]. The data shows this domain can form the required secondary and tertiary structure and maintain that structure as structural changes only started taking place at 44°C during thermal denaturation. The melting temperature of 67.94°C obtained in this study is also not substantially lower than that of the full-length VP6, which has a melting temperature of 78°C [58] and higher with additional stabilizing components. The domain stability was also evident in the chemical denaturant data as it only loses 50% of its structure in 4.5 M urea and 1.5 M guanidine hydrochloride, respectively. Studies on blue tongue virus (BTV) VP7, a RV VP6 structural homologue, show BTV-VP7 to retain its native structure even in 5 M urea [59]. By comparison, the VP6_H_ domain is less stable chemically compared to a full-length protein of a similar structure to VP6 which is expected due to the decrease in size compared to the full-length protein. The VP6_H_ comprises approximately 50% of the VP6 protein structure yet the stability parameters indicate it is only slightly less stable than the full-length protein.

VP6_H_ has been shown to be structurally an independent domain containing all the information necessary for trimer formation as well as generating viral capsid-type particles [39]. Our data indicates that, in the absence of the base domain (VP6_B_), VP6_H_ can still fold to produce secondary and tertiary structure equivalent to the full-length protein. Since irreversible folding intermediates are formed under equilibrium conditions, VP6_H_ does not show simple unfolded-to-folded transitions as for monomeric protein domains [60]. The intermediates formed are not the classical molten globule as the data indicates a loss of secondary structural elements before significant disruption of the tertiary structure (Figure 4), which is in direct contrast with the order for the molten globule [61]. The intermediate does not consistently appear with the addition or removal of urea to the same concentrations but is more consistently observed for GdCl. Both urea and GdCl are effective in disrupting the hydrogen bonding network that stabilize higher-order protein structures, with urea known to preferentially interact with hydrophobic regions in proteins, while GdCl readily masks electrostatic interactions. The unfolding pathways revealed by the respective denaturants therefore depend on the bonds and interactions that stabilize the native conformation [52–54,62–64]. The differences in the denaturant's behavior and the differences seen in the behavior of VP6_H_ indicate that cooperativity is lost due to changes in the interactions the domain can make. The VP6_H_ makes many contacts in forming a viral capsid layer. Key interactions the domain makes with the base domain and other VP6 monomers are important for driving specific conformational changes during the viral life cycle. When the rotavirus outer layer is lost on cell entry, the VP6 layer undergoes substantial rearrangements to initiate transcription via the VP2 layer contacts [65].

## Conclusion

Bacterially expressed VP6_H_ is purified following a simple solubilisation process using low urea concentrations which do not denture the protein. Pure VP6_H_ has native-like secondary and tertiary structure and is stable up to 44°C where structural changes start to take place forming an intermediate and finally irreversible aggregation. The presence of 2 M urea removes the intermediate and delays aggregation and unfolding to approximately 60°C. Chemical denaturation is reversible in GdCl but not in urea and intermediates are detected with both denaturants indicating the domain is not a cooperative independent folding unit but that interactions made with the base domain provide a critical contact network through which VP6 adjusts conformation during the viral life cycle.

## Supplementary Material

Supplementary Figures S1-S4

## Data Availability

Upon submission, authors agree to make any data and associated protocols available upon request.

## Abbreviations

*E. coli*: *Escherichia coli*
far-UV-CD: far-ultraviolet circular dichroism
GdCl: guanidine hydrochloride
RV: Rotavirus
VP: viral protein
VP6_H_: VP6 head domain
VP6_B_: VP6 base domain
