# Supplementary material for: Spectroscopic analysis of the bacterially expressed head domain of rotavirus VP6
Source: Biosci Rep. 2024 Apr 29;44(5):BSR20232178. doi: 10.1042/BSR20232178 (PMC11065646; doi:10.1042/BSR20232178)
Supplement: Supplementary Figures S1-S4 [file BSR-2023-2178_supp.pdf]

Spectroscopic Analysis of the Bacterially Expressed Head Domain of *Rotavirus* VP6

Milaan Simone STRACHAN[1], Tshepo MASHAPA[1], and Samantha GILDENHUYS [1*]

[1]Department of Life and Consumer Sciences, College of Agriculture and Environmental Sciences, University of South Africa, Private Bag X6, Florida, Roodepoort, South Africa, 1710.

* Corresponding author **E-mail: gildes@unisa.ac.za** **Tel**: 011 471 3295 **Fax**: 011 471 2796

Contributing authors E-mail:

Milaan Simone Strachan: **68622600@mylife.unisa.ac.za**

Tshepo Mashapa: **tmashat@unisa.ac.za**

Supplementary figures

Figure S1: Bacterial Expression of $VP6_H$.

Figure S2: Solubilisation of $VP6_H$.

Figure S3: Purification of $VP6_H$.

Figure S4: AlphaFold models aligned

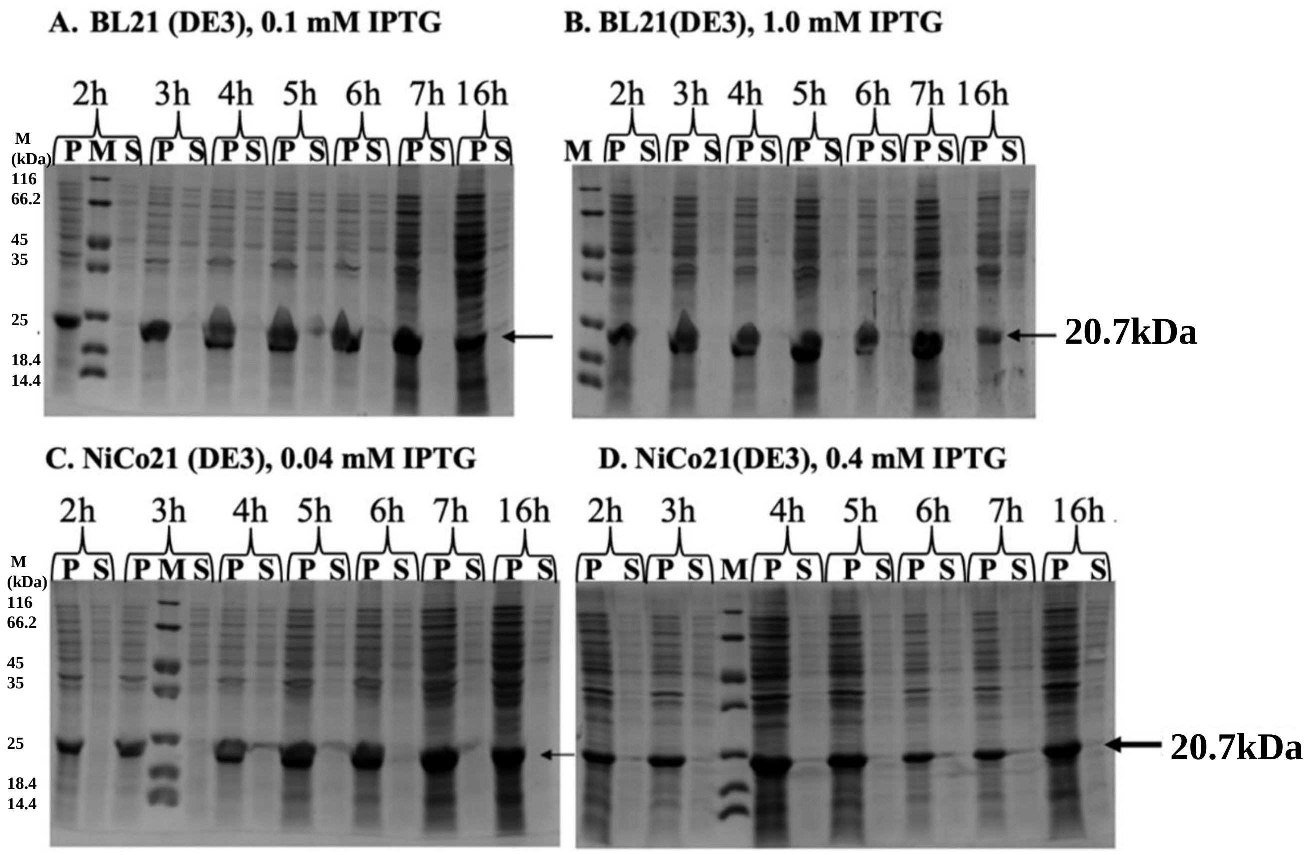

Figure S1: Bacterial Expression of VP6$_H$.
SDS-PAGE of VP6$_H$ expressed in the Bl21 (DE3) and NiCo21 (DE3) *E. coli* strains. Pellet (P) and supernatant (S) samples from cells grown for 2 h, 3 h, 4 h, 5 h, 6 h, 7 h, and 16 h post-induction are shown. A and B represent BL21 (DE3) cultures induced with 0.1 mM and 1 mM IPTG; C and D represent NiCo21 (DE3) cultures induced with 0.04 mM and 0.4 IPTG, respectively. M is the molecular weight marker, and the arrows indicate VP6$_H$ with the calculated relative molecular weight.

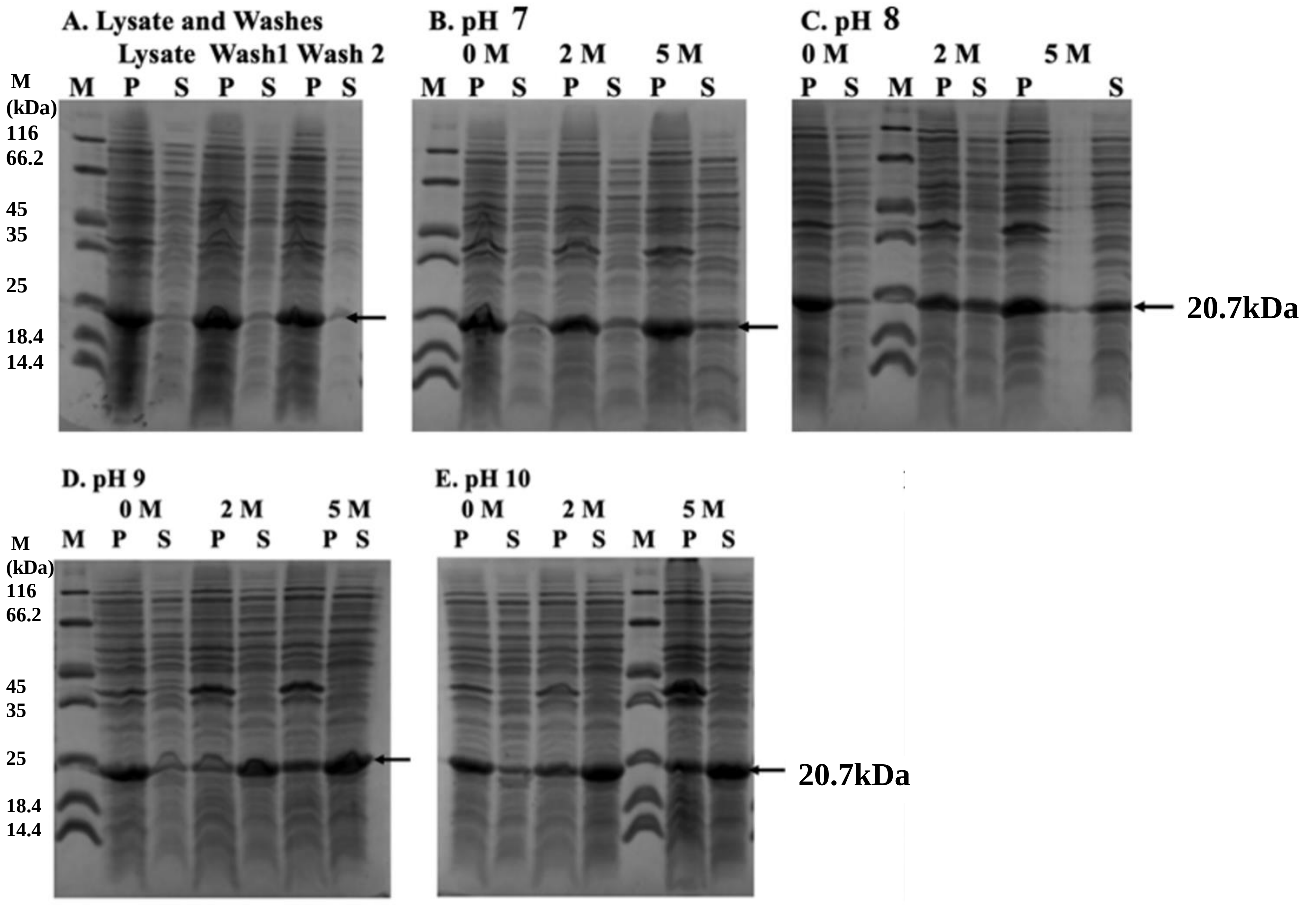

Figure S2: Solubilisation of $VP6_H$.
SDS-PAGE of $VP6_H$ frozen in 100 mM Tris-HCl buffers of varying pH (7 – 10) and urea concentrations (0 M, 2 M, and 5 M). Washes in buffers with 1 % Triton X-100 (Wash 1) and 0 % Triton X-100 (Wash2). Solubilisation at pH 7 (B), pH 8 (C), pH 9 (D) and pH 10 (E), with and without urea (2 or 5 M). Pellet (P) and supernatant (S) samples are indicated. M is the molecular weight marker and $VP6_H$ is indicated by the arrow with the calculated relative molecular weight.

A.

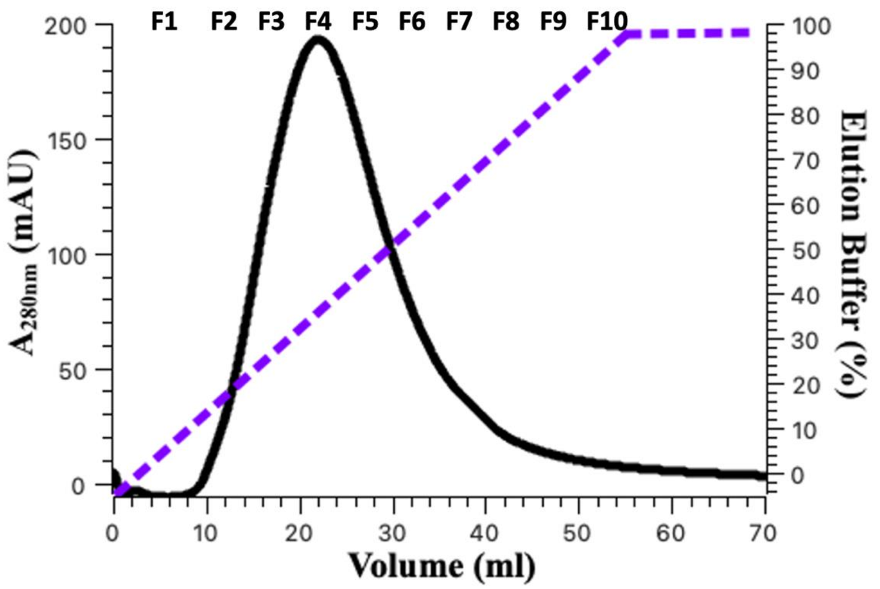

B.

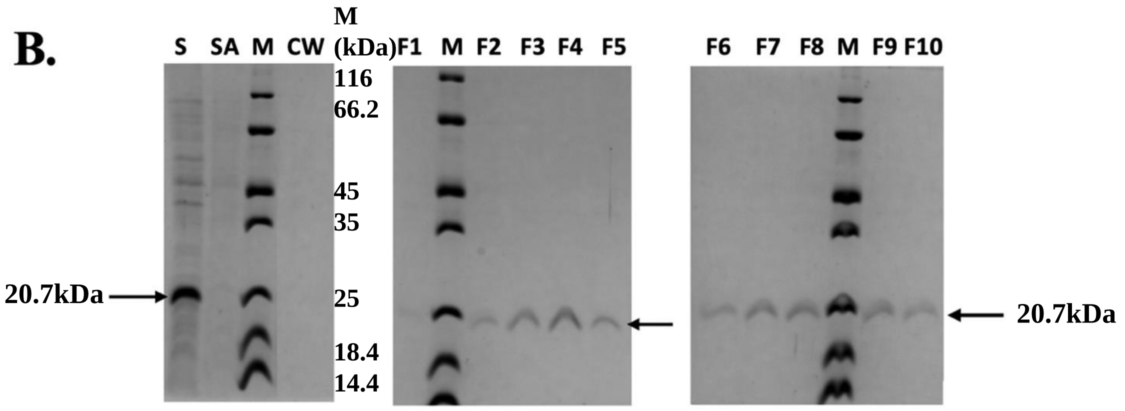

Figure S3: Purification of VP6$_H$.

(A)Elution profile of VP6$_{H.}$ The domain was eluted by an imidazole gradient (purple) using 100 mM Tris-HCl pH 9.00, 2 M urea, 300 mM NaCl, 40 mM imidazole, and 0.02% (w/v) sodium azide and an elution buffer of the same composition just with 600 mM imidazole instead of 40mM imidazole. (B) SDS-PAGE of the VP6$_H$ purification flow through and eluent. S is soluble VP6$_H$, SA is the sample application flow through, CW is the column wash flow through, F1 – F10 are the fractions collected, M is the molecular weight marker, and the arrows indicate VP6$_H$ with the calculated relative molecular weight.

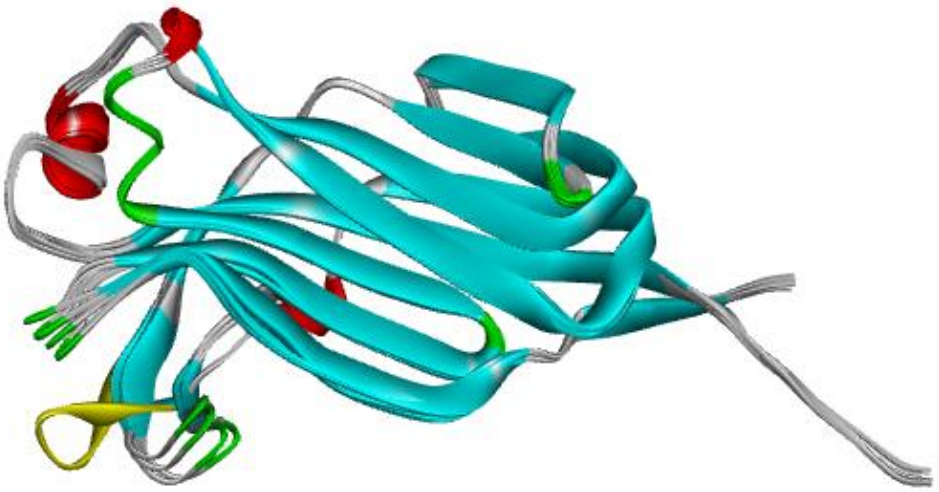

Figure S4: AlphaFold models aligned

The $VP6_{H.}$ domain with our consensus sequence and his-tag was modeled using AlphaFold. Five models were generated, one of which displays variation in the region Ile91-Trp100 (Ile238- Trp 247 in the full-length protein) displayed here in yellow. Image created using Discovery Studio client v20.1.0.19
